# Dairy Manure Co-composting with Wood Biochar Plays a Critical Role in Meeting Global Methane Goals

**DOI:** 10.1021/acs.est.2c03467

**Published:** 2022-07-14

**Authors:** Brendan P. Harrison, Si Gao, Melinda Gonzales, Touyee Thao, Elena Bischak, Teamrat Afewerki Ghezzehei, Asmeret Asefaw Berhe, Gerardo Diaz, Rebecca A. Ryals

**Affiliations:** † Environmental Systems Graduate Group, School of Engineering, University of California Merced, Merced, California 95343, United States; ‡ Department of Life and Environmental Sciences, School of Natural Sciences, University of California Merced, Merced, California 95343, United States; § Department of Mechanical Engineering, School of Engineering, University of California Merced, Merced, California 95343, United States

**Keywords:** composting, biochar, livestock, natural climate solutions, climate change mitigation, methane

## Abstract

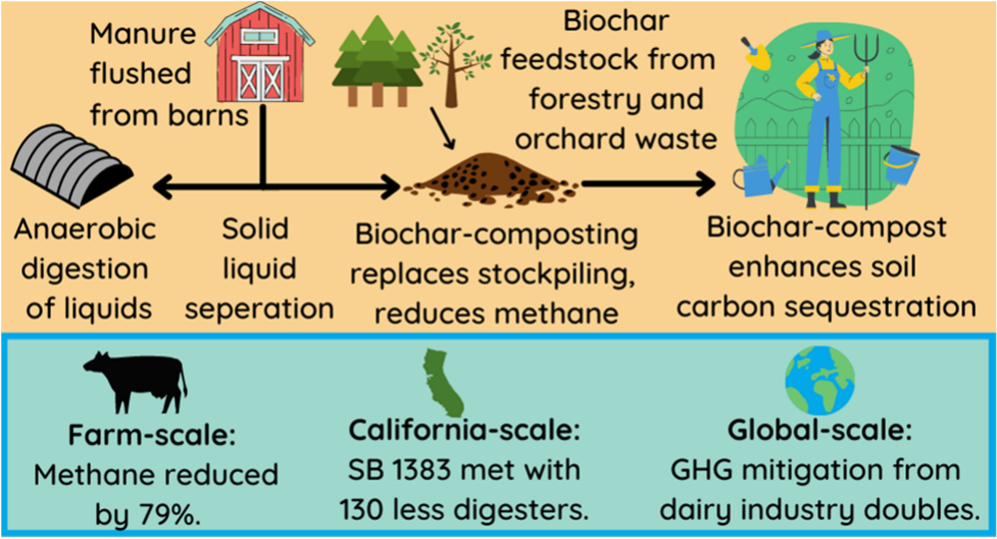

Livestock are the largest source of anthropogenic methane (CH_4_) emissions, and in intensive dairy systems, manure management can contribute half of livestock CH_4_. Recent policies such as California’s short-lived climate pollutant reduction law (SB 1383) and the Global Methane Pledge call for cuts to livestock CH_4_ by 2030. However, investments in CH_4_ reduction strategies are primarily aimed at liquid dairy manure, whereas stockpiled solids remain a large source of CH_4_. Here, we measure the CH_4_ and net greenhouse gas reduction potential of dairy manure biochar-composting, a novel manure management strategy, through a composting experiment and life-cycle analysis. We found that biochar-composting reduces CH_4_ by 79%, compared to composting without biochar. In addition to reducing CH_4_ during composting, we show that the added climate benefit from biochar production and application contributes to a substantially reduced life-cycle global warming potential for biochar-composting: −535 kg CO_2_e Mg^–1^ manure compared to −194 kg CO_2_e Mg^–1^ for composting and 102 kg CO_2_e Mg^–1^ for stockpiling. If biochar-composting replaces manure stockpiling and complements anaerobic digestion, California could meet SB 1383 with 132 less digesters. When scaled up globally, biochar-composting could mitigate 1.59 Tg CH_4_ yr^–1^ while doubling the climate change mitigation potential from dairy manure management.

## Abstract 2

We show that dairy manure biochar-composting could offer governments an untapped, powerful, and cost-effective tool to help achieve global methane reduction targets by the end of the decade.

## Introduction

Agriculture is responsible for one-third of emissions of global greenhouse gas (GHGs), and methane (CH_4_) from agriculture accounts for 35% of food-system emissions.^1^ Livestock are the leading source of anthropogenic CH_4_. In developed countries, the industrialization of animal agriculture has concentrated emissions, pollution, and manure into feedlots with relatively small physical footprints.^2−4^ Dairy feedlots in particular present a significant nutrient recycling and GHG mitigation opportunity due to their large stocking densities, high rate of manure production, and spatial decoupling of livestock from feed production.^5,6^ Optimizing the treatment and reuse of dairy manure could help prevent nutrient loss while substantially reducing CH_4_ emissions. This is especially relevant in dairy-intensive regions such as California, where dairy manure accounts for 25% of total CH_4_ emissions.^6−9^

In 2016, California enacted SB 1383, which requires CH_4_ from dairies to be reduced by 40% below 2014 levels by 2030.^10^ More recently, The Global Methane Pledge, which was signed by 110 countries at the Conference of the Parties 26 (COP26), calls for a 30% reduction in CH_4_ from 2020 levels by 2030.^11^ While most Global Methane Pledge signatories do not yet have detailed plans for reducing CH_4_ from manure, California plans to meet its dairy CH_4_ reduction goal primarily through the deployment of anaerobic digesters, which capture CH_4_ from liquid manure lagoons for energy production.^8^ However, California is not currently on track to meet this goal, in part due to economic barriers to constructing anaerobic digestion systems.^12,13^ Additionally, CH_4_ mitigation at dairies with digesters is limited to the liquid portion of manure, while separated solids are often stockpiled in large, static piles favorable for CH_4_ production.^6,14,15^ An effective strategy to reduce CH_4_ from separated solid manure could increase the CH_4_ mitigation potential for every digester installed and reduce the number of digesters needed to achieve CH_4_ reduction goals.

One alternative to stockpiling is aerobic composting. While composting can reduce CH_4_, eliminate pathogens, and create a valuable soil amendment, it can still be a significant source of nitrous oxide (N_2_O) and CH_4_.^16−18^ Some studies have shown that when added to compost, biochar, a carbon-rich material produced through biomass pyrolysis, can reduce GHG by improving aeration, adsorbing gases, and stimulating key microorganisms.^19−22^ Combining biochar with organic waste is an agricultural technique that is practiced by numerous Indigenous Peoples, but only a few studies have quantified the GHG benefit of biochar-composting at the field-scale, and none has used separated dairy manure solids as a feedstock.^23−26^ It also remains unclear whether scaling up this technology can play a significant role in meeting CH_4_ mitigation goals.^10,27^

Here, we conducted a field-scale composting experiment to measure GHG emissions during the composting of separated solid dairy manure with and without biochar. We hypothesize that CH_4_ is reduced from biochar-amended piles due to improved pile aeration.^20^ GHG results from the composting experiment are incorporated into a life-cycle assessment (LCA) of solid manure management systems. Finally, LCA results are used to estimate the role that biochar-composting can play in meeting CH_4_ reduction goals in California and globally.

## Materials and Methods

### Site Description and Experimental Setup

The composting experiment was conducted at Philip Verwey Dairy in Madera, California (36°56′03″N, 120°23′09″W), from Aug to Sept 2021. The biochar used in the experiment was Rogue Biochar from Oregon Biochar Solutions (White City, OR). Biochar feedstock was composed of approximately 85% Douglas fir (*Pseudotsuga menziesii* L.) and Ponderosa pine (*Pinus ponderosa* L.) wood waste mixture, 14–15% almond and walnut tree pruning, and <1% nutshells. The maximum pyrolysis temperature was reported to be 900 °C (K. Strahl, pers. comm.). The characteristics of biochar, composts, and compost feedstocks are listed in Supporting Tables 1 and 2.

A manure-only compost windrow pile and a biochar-compost windrow pile were prepared on-site on Aug 10th, 2021. Each pile was trapezoidal in shape and approximately 30 m in length, 3 m in width, and 1 m in height. The manure-only pile consisted of approximately 15.34 t fresh solid manure and 1.32 t orchard clipping residues (3.37 t dry manure and 1.2 t dry clipping residues). Biochar-compost pile consisted of 15.35 t fresh solid manure, 1.32 t orchard clipping residues, and 1.0 t biochar (3.37 t dry manure, 1.2 t dry clippings, and 0.91 t dry biochar). Both piles were turned weekly a total of 4 times (on days 8, 15, 22, and 29) throughout the 35-day experiment.

### Greenhouse Gas Flux Measurement and Compost Characterization

Compost greenhouse gas fluxes (CO_2_, N_2_O, and CH_4_) were measured daily over the 35-day experiment using a cavity ring-down laser spectrometer (Picarro G2508, Picarro Inc., Santa Clara, CA) connected to a closed system static chamber (made from polyvinyl chloride) and 26 cm diameter by 13 cm tall). Collars (made from polyvinyl chloride) and 25.5 cm diameter by 15 cm tall) were inserted 3 cm into the compost pile and allowed to sit for 1 h before measurement. Gas was sampled daily from nine locations (three South side, three top, and three North side) on each windrow, as shown in Supporting Figure 1, by fitting the chamber lid over a collar (creating a total chamber volume of 12 271.9 cm^3^) and sampling for 5 min. After taking a measurement, gas concentrations were allowed to return to ambient concentrations before the next measurement. Gas fluxes (nmol m^–2^ s^–1^) were calculated in the Picarro Soil Flux Processor program using the exponential model developed by Hutchinson and Mosier to account for nonlinear changes in headspace concentration.^28^ To account for the “chimney effect” and the spatial variation within the pile, we considered each pile’s dimensions when calculating gas fluxes and emission factors at the scale of the compost pile following Andersen et al. and Sánchez et al.^29,30^ Specifically, the average pile flux was calculated as (North side flux × North side surface area + top side flux × top side surface area + South side flux × South side surface area)/compost pile base area. Surface area and base area were measured and estimated weekly to ensure that the temporal changes in pile dimensions were taken into account over the course of the experiment. The average pile fluxes (nmol m^–2^ s^–1^) were later converted to daily emission factors presented as g or mg trace gas or C or N kg^–1^ dry feedstock d^–1^ using the feedstock mass data.

For each flux measurement, compost surface temperature was measured with a digital probe thermometer (PDT650, UEi Test Instruments, Indianapolis, IN), and chamber temperature was measured with a suction cup thermometer (Taylor Precision Products, Oak Brook, IL) attached to the top of the chamber. Pile temperature was measured daily by inserting two 5TE sensors connected to an EM50 data logger (METER Group, Pullman, WA) into the center of the pile approximately 30 cm deep at a height of 30 cm and allowing the temperature to stabilize over at least 1 h.

Fresh compost samples were collected weekly after piles were turned to determine physiochemical properties. Briefly, compost moisture content was determined by weighing the fresh and dried sample before and after drying in an oven at 105 °C for 24 h. Compost pH was determined in 1:2 sample to DI water (v/v) suspension. Porosity was determined following the protocols described in Flint and Flint.^31^ Compost NH_4_^+^-N and NO_3_^–^-N concentrations were determined by shaking 3 g of compost in 30 mL of 2 M KCl and analyzing extractions on a Lachat QuikChem 8500 Flow Injection Analyzer (Lachat Instruments, Milwaukee, WI). Total C and N were analyzed on oven-dried samples (105 °C) using an elemental analyzer (Costech 4010, Costech Analytical Technologies Inc., Valencia, CA). The compost germination index was determined according to Luo et al.^32^

Initial biochar and manure feedstocks were analyzed for total C, total N, pH, NH_4_^+^-N, and NO_3_^–^-N using the same methods used for compost. Proximate analysis of feedstocks and compost was also conducted following ASTM D3172-13.^33^ Biochar surface area and pore characteristics were determined using the Brunauer, Emmett, and Teller (BET) method on a TriStar II Plus (Micromeritics, Norcross, GA).^34^ Biochar surface images at 50× and 200× magnification were taken using a scanning electron microscope (Supporting Figure 2) (Gemini500 FE-SEM, ZEISS, Dublin, CA).

### Statistical Tests

All statistical analyses were performed using the open-source statistical analytical software R. Cumulative gas emissions were estimated by calculating the area under the daily emission curves using the function auc() in package “flux” in R.^35,36^ Analysis of variance (ANOVA) and Tukey’s post hoc tests were carried out on weekly cumulative gas emissions to examine the significance of biochar treatment at *P* = 0.05. Pearson correlation tests were conducted on selected variables that were of interest to us to elucidate the relationships between gas emissions and compost characteristics throughout the experiment. In addition, we used a mixed linear regression (MLR) model to determine the dominant drivers controlling gas fluxes in our 35-day field study (Supporting Table 3). All data were tested for homogeneity of variance and normality of residuals before the MLR analyses and were log-transformed when necessary.

### Life-Cycle Assessment

A life-cycle assessment (LCA) was conducted to estimate the climate impacts associated with each major stage of biochar-composting, composting, and stockpiling.^17^ The functional unit for the model is one metric ton of separated solid dairy manure, and we use manure stockpiling as a reference system to account for avoided emissions. The LCA system boundary begins with raw feedstock transportation and ends with compost application to the soil. While we account for the portion of the carbon in each amendment that is likely to remain stable in soil long-term, we exclude ecosystem impacts from amendment application due to the lack of field studies that measure changes in soil GHG fluxes and plant biomass after the application of biochar-compost to the soil, but improvements to the model can be made when this data becomes available.^37−41^ Both the 20-year and 100-year global warming potentials (GWPs) were quantified for each system. A 20-year global warming potential was included because CH_4_ has a high GWP over its short 12-year lifespan, which is relevant in the context of CH_4_ reduction policies like SB 1383 and the Global Methane Pledge that are designed to help mitigate the most devastating impacts of climate change over the next few decades as governments begin to transition away from fossil fuels.^2,8,42,43^ Biogenic CO_2_ emissions from composting are assumed to be climate neutral in our primary model because the carbon originates from recently photosynthesized CO_2_ and has no net climate impact,^17,44,45^ although we do include a model version that accounts for biogenic CO_2_ emissions in the supporting material (Supporting Figure 3a).

In our LCA, we use our experimental cumulative GHG fluxes for the composting and biochar-composting stages. To estimate GHG fluxes from stockpiling, we use our compost emission data and assume an average reduction in CH_4_ and N_2_O by 71 and 50%, respectively, when manure is composted instead of stockpiled.^18^ The portion of C in compost that can be sequestered in soil long-term is assumed to be 9%, which is the mid-range value presented in a review by Martínez-Blanco et al.^38^ We use a 97% C sequestration rate for the biochar fraction of biochar-compost, which is based on the results of a meta-analysis by Wang et al.^37^ Avoided fossil fuel emissions from the energy produced from pyrolysis are estimated using a net energy production value for pyrolysis of 4043 MJ/feedstock and a 28.8% biochar yield from Roberts et al. as well as IPCC default emission factors for coal and natural gas (assuming a 50/50 mix in the baseline scenario).^46,47^ We assume that gases produced during pyrolysis are combusted and the only GHGs released are biogenic CO_2_.^46^ Biochar production can also reduce GHG emissions from biomass burning of crop residues and forestry waste, and we assume that 10% of the woody feedstock used in composting and biochar production would have otherwise been burned.^48−50^ This value is based on the percent of wheat and corn residues (the two largest sources of crop residues in the U.S.) burned in the United States annually.^51,52^ Feedstock and composts are transported by 36-ton diesel trucks and are distributed locally (5–40 km) in each strategy’s baseline scenario.^53,54^

### Sensitivity and Uncertainty Analyses

Since the LCA model has nonlinearities, we performed a global sensitivity analysis.^55^ First-order (S1) indices measure the singular effect of a parameter on variance in the output, and total-order (S.T.) indices measure the total effect or first- and higher-order effects (multiplicative effects) of a parameter.^56^ We used a variance-based Sobol analysis method, given its easy computation and interpretation.^56^ The ranges applied to each variable are shown in Supporting Table 4. The LCA was first programmed in Python, and the experiment was performed using the SALib library.^57^ Samples (2048) were generated from the given parameter space (Supporting Table 4) using a Saltelli sampler. This number of samples was enough to ensure convergence in the index’s values. From the sensitivity analysis experiment, we analyzed the output space from each management strategy to characterize their uncertainty.

### Methane Reduction from Biochar-Composting in California

For our California analysis, we estimate the number of additional anaerobic digesters needed to meet California’s 40% CH_4_ reduction goal without biochar-composting (digester + stockpiling scenario), with biochar-composting (digester + biochar-composting scenario), and with biochar-composting along with a 1% annual decrease in statewide herd population (enhanced population reduction scenario). The average annual CH_4_ reduction rate per digester was calculated according to IPCC Tier 2 guidelines.^58^ We used California specific values for the average number of lactating cows per dairy and the mass of volatile solids produced per head, as well as the maximum methane production capacity (*B*_0_) and methane conversion factors (MCFs) for both anaerobic lagoons and anaerobic digesters^59^ (Supporting Table 5). We account for the CH_4_ emissions avoided from converting an anaerobic lagoon into an anaerobic digester, as well as the direct CH_4_ emissions from anaerobic digesters due to leakages and incomplete combustion.^59,60^ The net dairy manure CH_4_ reduction associated with biochar-composting was calculated by subtracting the CH_4_ emitted during biochar-composting from the CH_4_ avoided by not stockpiling, which are both taken from our LCA.

CH_4_ reductions for anaerobic digestion and biochar-composting are relative to a baseline system similar to the model in Owen and Silver in which dairy manure from mature and lactating cows is separated into a solid fraction, which is stockpiled, and a liquid fraction, which is stored in an anaerobic lagoon.^5^ We assume a 50% solid separation rate, which is the average efficiency of the four solid–liquid separation technologies reviewed in Hjorth et al.^61^ We do not consider any manure managed from heifers or calves because manure from immature and nonmilking cows is typically managed through alternative methods such as daily spread or dry lot systems that yield little CH_4_, and according to an analysis by Marklein et al., account for less than 2% of total dairy CH_4_ emissions.^9^ Current progress on SB 1383, which we use as a baseline in our model, is based on a recent CARB report that estimates that by 2022, the state will have reduced dairy CH_4_ emissions by 3.5 MMT and will have 130 anaerobic digestors operating.^8^ Our model also accounts for CH_4_ reduction contributions from CARB projected reductions in statewide herd population (0.5% in digester + stockpiling and digester + biochar-composting scenarios and 1% in enhanced population reduction scenario), as well as CARB projected increases in the number of other alternative manure management projects (assuming a rate of 1 AAMP project implemented per digester project) likely to be done at smaller farms unable to install anaerobic digesters.^8^ We incorporate the CH_4_ mitigation from annual herd population reduction by assuming that the 40% CH_4_ reduction goal is achieved in 2030 in each scenario, and the total CH_4_ savings from herd population reduction from 2022 to 2030 are distributed equally over the number of new digesters built from 2022 to 2030. A 100-year GWP is used in this estimate because the state’s goal of 9 MMT CO_2_e reduction in CH_4_ is based on a 100-year CH_4_ conversion factor.

### Global GHG Mitigation Potential of Biochar-Composting

For our global analysis, we quantify the total GHG and CH_4_ mitigation of anaerobic digestion and biochar-composting dairy manure management systems when scaled up to their global potential. Like Höglund-Isaksson et al., we assume that it is only economically feasible to install anaerobic digesters at dairies with herd sizes greater than 100 head.^2^ An estimate of the number of dairy cattle kept on farms with greater than 100 head is taken from Höglund-Isaksson et al.^2^ We then calculate a per head GHG mitigation rate for anaerobic digestion and biochar-composting and assume a 50% solid–liquid separation efficiency.^61^ The annual mass of manure volatile solids produced per head is estimated using average values for total animal mass and volatile solids produced per total animal mass from North America, Europe, and Asia, the regions most likely to be suitable for anaerobic digestion projects due to their intensive dairy systems (Supporting Table 5).^2,58^ IPCC default emission factors are used to estimate anaerobic digestion CH_4_ reduction per head, and we use Environmental Protection Agency (EPA’s) guidelines for estimating the avoided fossil fuel emissions from biogas electricity production.^58,62^ Emission reductions from biochar-composting are taken from our LCA results, and we consider different rates (0–100%) of on-farm biochar-composting where it is assumed that any manure solids not biochar-composted are stockpiled.

## Results and Discussion

### Dairy Manure Biochar-Composting Experiment

**Figure 1:**
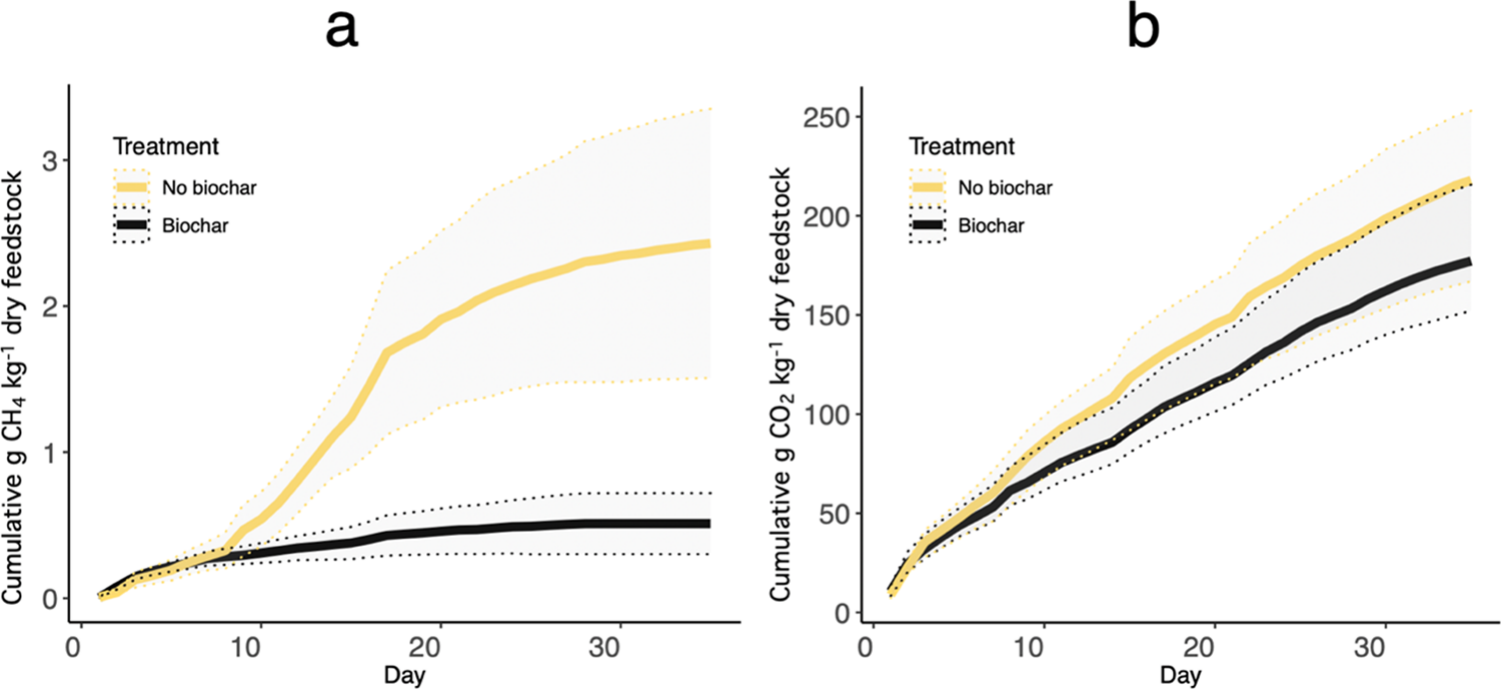
Cumulative CH_4_ (a) and CO_2_ (b) emissions over the 35-day composting experiment. CH_4_ emissions are expressed in units of g CH_4_ kg^–1^ dry feedstock. CO_2_ emissions are expressed in units of g CO_2_ kg^–1^ dry feedstock. The black curve shows cumulative emissions from biochar-composting, and the yellow curve shows cumulative emissions from composting. The shaded region for each curve shows the 95% confidence interval for each pile’s gas flux measurements.

We conducted a 35-day field-scale composting experiment to measure differences in daily GHG fluxes during the composting of dairy manure solids amended with or without biochar. Over the course of the experiment, the manure-only pile emitted 2.43 g CH_4_ kg^–1^ dry feedstock, 218 g CO_2_ kg^–1^ dry feedstock, and 0.029 mg N_2_O kg^–1^ dry feedstock (Figure 1, Supporting Figure 4). In contrast, cumulative emissions from the biochar-compost pile were 0.51 g CH_4_ kg^–1^ dry feedstock, 177 g CO_2_ kg^–1^ dry feedstock, and 0.075 mg N_2_O kg^–1^ dry feedstock (Figure 1, Supporting Figure 4).

Differences in cumulative CO_2_ and N_2_O emissions for each pile were not statistically significant (Figure 1; Supporting Figure 4). Similar to Vergara and Silver, both piles had very low N_2_O fluxes, which may be due to low initial nitrate (NO_3_^–^) concentration in both composts (Supporting Figure 5) as well as potential nitrification inhibition from the high temperatures maintained throughout the composting experiment.^17^

There was a significant reduction in CH_4_ emissions when biochar was added to the composting process (*P* > 0.001, Figure 1). The biochar-compost CH_4_ emission factor was 79% less than that of the manure-only pile. The majority of CH_4_ was emitted during the first 3 weeks of composting for both piles (81% for manure-only and 91% for biochar-compost; Supporting Figures 6 and 7), which is consistent with other manure composting studies.^14,17^

We find that CH_4_ mitigation in the biochar-compost pile is highly correlated with moisture content, which was significantly lower than in the manure-only pile (Supporting Figures 8, 9 and Supporting Table 3). This is consistent with previous findings that suggest adding biochar to compost can decrease CH_4_ emissions by increasing pile aeration and O_2_ diffusion due to biochar’s high micro and macroporosity.^19,20^ An increase in O_2_ from biochar addition could reduce CH_4_ production by methanogens and increase CH_4_ consumption by methanotrophs, reducing the net CH_4_ flux from the biochar-compost pile.^19,20,63^ Biochar could have advantages over other compost bulking agents because it provides very little labile C compared to biomass that has not been pyrolyzed and labile C can drive CH_4_ emissions. The biochar may also reduce CH_4_ emissions through the adsorption of manure labile C and CH_4_ during composting.^21^ Though the biochar-compost pile had a higher pH than the manure-only pile, the pH values of both composts were in the range suitable for methanogenesis (Supporting Table 1).^64^ Additional studies are needed that isolate and investigate other potential biological and physicochemical mechanisms through which biochar could mitigate composting CH_4_ emissions.^20^

Excluding turning days when the compost temperature dropped by 5–10 °C for approximately 24 h, the temperature for both piles ranged from 65 to 73 °C (Supporting Figure 8). While the biochar-compost pile reached peak temperature faster, there was no significant biochar treatment effect for temperature throughout the experiment (*P* > 0.10). The biochar-compost pile had a lower moisture content than the manure-only pile throughout the experiment, and the moisture content of both piles dropped by week 5 (Supporting Figure 8). Both composts reached maturity at the end of week 5, which was demonstrated by a germination index above 50, NH_4_^+^-N less than 0.4 g/kg, and a C/N ratio less than 25 (Supporting Table 1).^65^ While the composting process can be done over a much longer period, we found that 35 days were suitable for our compost to reach maturity and be suitable for use as a soil amendment in agroecosystems. Shorter composting times are likely needed in intensive dairy systems that have high daily rates of manure production and limited space for composting given that compost maturity indices are met, and compost temperatures reach a minimum of 55 °C for 3 days as required by the USDA.^65−67^

While our study shows that biochar-composting has substantial CH_4_ mitigation potential when implemented in dairy systems, our experiment used only one type of biochar applied at a single rate. Biochar physiochemical properties can vary greatly depending on the initial feedstock used and on the temperature and duration of pyrolysis. Different biochars applied at different rates may thus result in different capacities for biochar-composting to mitigate GHG emissions from dairy manure. For example, Pascual et al. found that soils amended with different types of biochars had different rates of CH_4_ emissions, likely due to differences in the physiochemical properties of the biochars.^68^ Therefore, research is needed that tests multiple types of biochars, applied at multiple rates, to optimize biochar feedstocks and application rates for the greatest GHG reduction during biochar-composting. This would allow researchers to make specific recommendations to dairy farmers interested in adding biochar to their manure compost.^25^

### Life-Cycle Assessment

**Figure 2:**
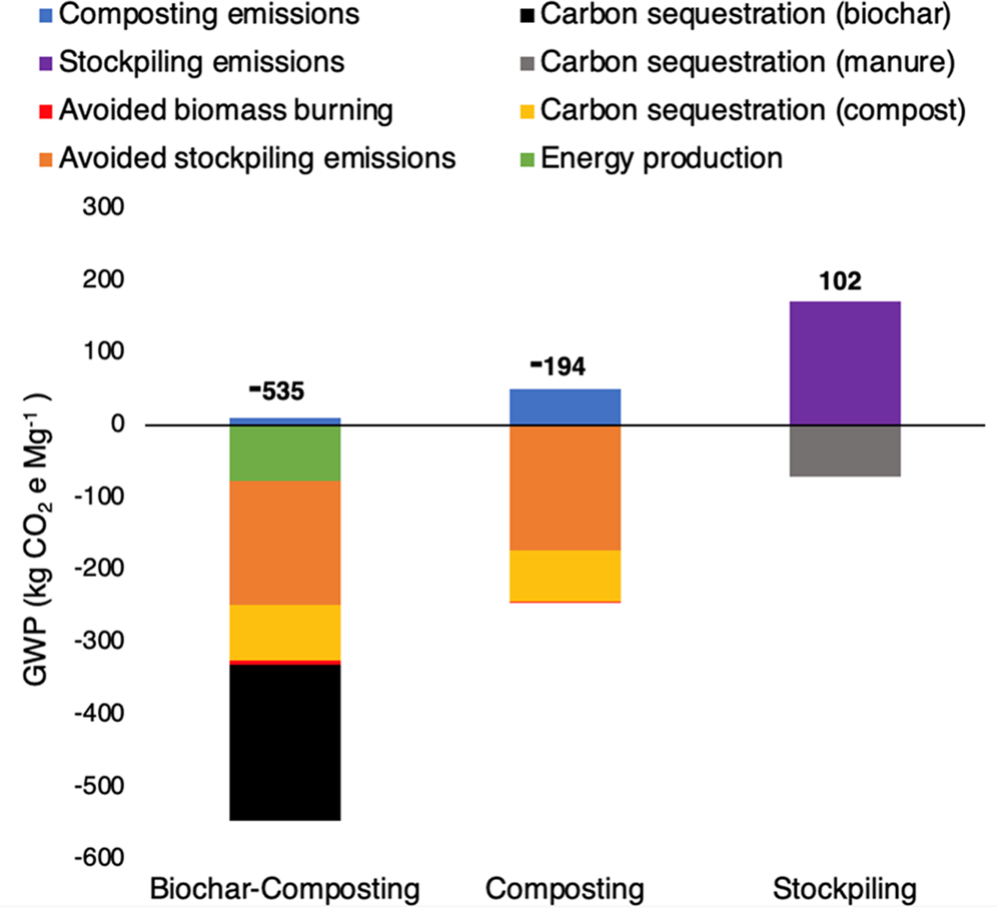
Life-cycle assessment of management strategies for separated solid dairy manure using 100-year GWPs. The number above each strategy is the net GWP in kg CO_2_e Mg^–1^ manure. Each color represents a different life-cycle stage and is referenced in the legend above. The transportation stages are removed from the figure due to their minuscule contribution to the total GWP of each strategy.

We incorporated our GHG data from the composting experiment into an LCA of solid dairy manure management strategies. Results show a significant reduction in net global warming potential (GWP) when a functional unit of one metric ton of fresh solid dairy manure is managed through composting or biochar-composting compared to a reference system in which separated solid manure is stockpiled (Supporting Figure 10). Results from our 100-year GWP model are −535 kg CO_2_e, −194 kg CO_2_e, and 102 kg CO_2_e for biochar-composting, composting, and stockpiling, respectively (Figure 2). Using a 20-year GWP, which some argue is appropriate when considering CH_4_ mitigation policies designed to reduce warming over the next few decades, the net climate impact of biochar-composting, composting, and stockpiling is −870 kg CO_2_e, −441 kg CO_2_e, and 446 kg CO_2_e, respectively (Supporting Figure 3b).^42,43^

The life-cycle stage with the largest reduction in GWP for biochar-composting and composting is the avoided CH_4_ emissions that would have occurred if the manure had been stockpiled. The largest source of emissions for biochar-composting and composting systems is direct composting emissions; however, biochar-composting had lower direct emissions (10 kg CO_2_e) compared to composting (50 kg CO_2_e) due to a 79% reduction in CH_4_ emissions (Figure 1). Compost carbon (C) sequestration from soil application is a large sink of emissions for composting (−71 kg CO_2_e) and biochar-composting (−77 kg CO_2_e), but the persistent biochar C in biochar-compost resulted in the additional sequestration of −215 kg CO_2_e (Figure 2; Supporting Figure 11). The avoided fossil fuel emissions from the electricity generated through biochar production also reduced the GWP of biochar-composting by −76 kg CO_2_e (Figure 2). When using a 20-year GWP and accounting only for direct emissions from each system by excluding the avoided emissions from stockpiling, biomass burning, and fossil fuel displacement, biochar-composting remains a net sink of emissions (−261 kg CO_2_e), while compost becomes a net source (79 kg CO_2_e) (Supporting Figure 12a).

Our LCA suggests that adding biochar to compost can enhance CH_4_ mitigation from solid dairy manure management systems while offering co-benefits such as electricity production, soil C sequestration, and sustainable woody biomass management. Unlike composting, biochar-composting has a negative GWP when excluding avoided emissions in the 20-year GWP model (Supporting Figure 12b). This is a significant finding as we work toward managing agroecosystems to function as a net sink of GHGs rather than a source. While our study is the first to use LCA to examine the climate change impact of biochar-composting as a solid dairy manure management strategy, our analysis is limited in that it does not include direct measurements of stockpiling emissions or agroecological impacts (e.g., changes in crop biomass or soil N_2_O fluxes) from compost and biochar-compost when it is used as a soil amendment. To better quantify the GWP of biochar-composting compared to other manure management systems, future studies are needed that examine the long-term climate change and agronomic impacts associated with the addition of dairy manure biochar-composts to the soil. Studies are especially needed that compare biochar-composting to other soil amendments or compare biochar-composts with different biochar feedstocks or biochar application rates.

### Sensitivity and Uncertainty Analyses

Results from a global sensitivity analysis show that the net GWP of each management strategy is most sensitive to parameters that affect the net CH_4_ output, such as CH_4_ GWP and CH_4_ emission factors for manure stockpiling and composting (Supporting Figure 13). Our uncertainty analysis shows that stockpiling manure almost always results in a net source of emissions, with a minimum of −6.62 kg CO_2_e Mg^–1^ manure and a maximum of 684.26 kg CO_2_e Mg^–1^ manure (Supporting Figure 14). Composting always results in a net sink with a minimum of −618.60 kg CO_2_e Mg^–1^ manure and a maximum of −96.16 kg CO_2_e Mg^–1^ manure. Biochar-composting almost always results in the largest net sink with a minimum of −920.87 kg CO_2_e Mg^–1^ manure and a maximum of −443.99 kg CO_2_e Mg^–1^ manure.

### Methane Reduction from Biochar-Composting in California

**Figure 3:**
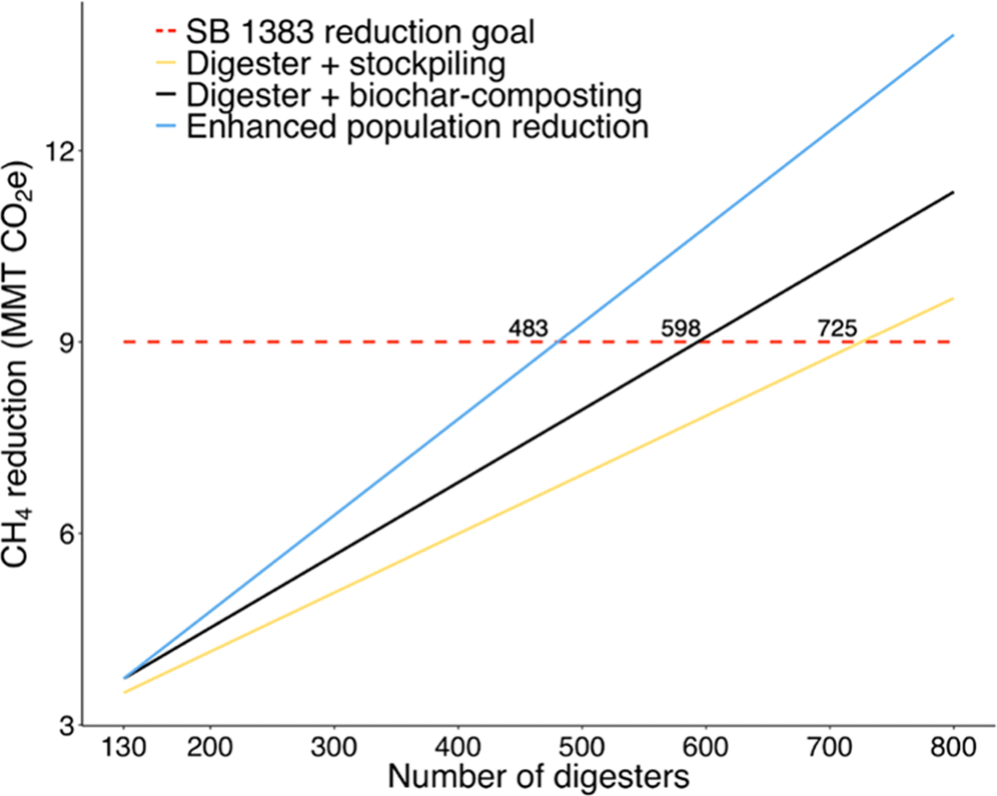
Number of anaerobic digesters needed to meet California’s 40% dairy CH_4_ reduction goal mandated by SB 1383 under different scenarios. The digester + stockpiling scenario assumes that dairies with anaerobic digesters stockpile their separated solid manure. The digester + biochar-composting scenario assumes that dairies with anaerobic digesters biochar-compost their separated solid manure. The enhanced population reduction scenario assumes that dairies with anaerobic digesters biochar-compost their separated solid manure and that the statewide herd population declines at an annual rate of 1% instead of the California Air Resources Board’s (CARB) projected annual reduction of 0.5%, which is used in the other scenarios. In addition to assumed population reduction rates, each scenario assumes CARB projected rates for implementing new alternative manure management projects at dairies not large enough for anaerobic digesters.

California aims to meet its 40% dairy methane reduction goal primarily using anaerobic digesters, but the state is currently not on track to meet this target.^8^ We estimate the role that biochar-composting could play in reducing CH_4_ when it is used to manage solid dairy manure separated from anaerobic digester systems in California (Figure 3). Average per farm CH_4_ reduction is estimated using our LCA results for biochar-composting and using IPCC Tier 2 guidelines with California specific values for anaerobic digestion.^58,59^ We also assume a 0.5% annual reduction in dairy cow population, which the California Air Resources Board (CARB) projects for 2022–2030, and account for additional Alternative Manure Management Projects that would be implemented on farms not eligible for anaerobic digesters.^8^

Our model shows that total CH_4_ mitigation on farms with existing anaerobic digesters increases by 29% when biochar-composting replaces solid manure stockpiling. The additional CH_4_ mitigation in the digester + biochar-composting scenario allows the state to meet its CH_4_ goal with 598 digesters, or 132 fewer digesters, than it would take in the digester + stockpiling scenario, a number nearly equivalent to the 130 digesters currently built or cited in California.^8^ However, the EPA’s AgSTAR anaerobic digester program has identified only 799 dairy farms that would be suitable for anaerobic digester projects in California because it is not economically feasible for smaller dairies to build digesters.^62^ Under the digester + stockpiling scenario, our model shows the state needing 91% of eligible dairy farm owners to build anaerobic digestion systems on their farms to meet SB 1383. Even under the digester + biochar-composting scenario, 74% of eligible dairies would need digesters. While anaerobic digestion could provide an additional revenue stream for dairy farmers, there are high upfront costs associated with installing digesters. High adoption rates may, therefore, be unlikely without additional funding in programs that reduce financial risk for farmers.^8^ Under our enhanced population reduction scenario, which includes biochar-composting and increases the current annual rate of dairy cow population reduction from 0.5 to 1%, California can meet SB 1383 with 483 digesters or a 60% adoption rate. This additional population reduction could allow California to meet its CH_4_ goal without having to rely on the high digester adoption rates required under the digester + biochar-composting or digester + stockpiling scenarios.

### Global GHG Mitigation Potential of Biochar-Composting

**Figure 4:**
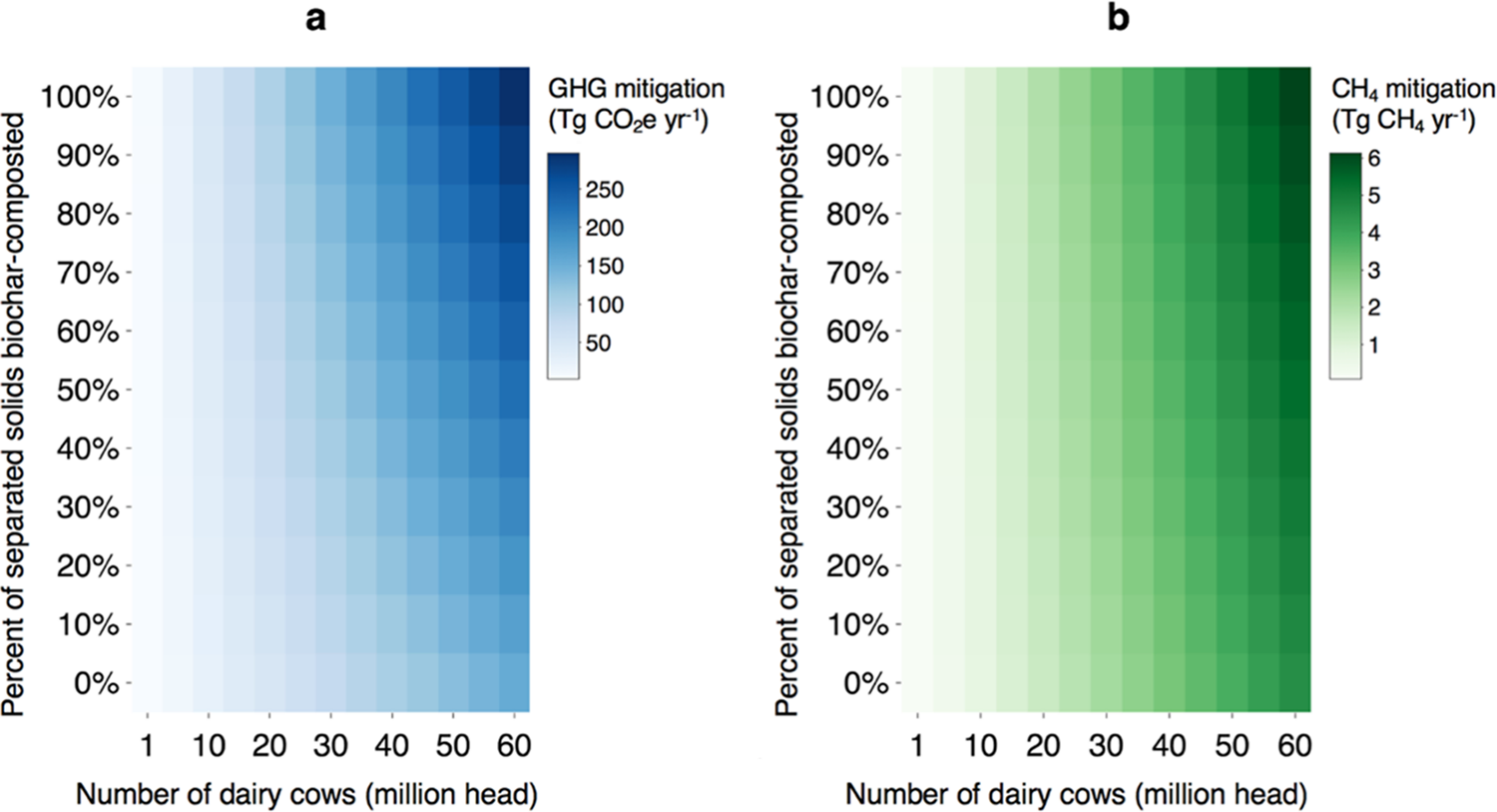
Technical GHG and CH_4_ global mitigation potential from dairy manure management. (a) Net life-cycle GHG mitigation from dairy manure management consists of anaerobic digestion of dairy manure and varying degrees of biochar-composting of separated solid manure. (b) CH_4_ mitigation from dairy manure management consists of anaerobic digestion of dairy manure and varying degrees of biochar-composting of separated solid manure. For each figure, the *x*-axis shows the hypothetical number of dairy cows (in million heads) managed in systems with anaerobic digesters. We limit our analysis to the number of dairy cows kept in intensive systems globally. The *y*-axis shows the percent of solid manure separated from digesters that is managed through biochar-composting. Solid manure that is not biochar-composted is assumed to be stockpiled.

We estimate the maximum technical CH_4_ and net GHG mitigation potential of biochar-composting when it is added to anaerobic digestion systems at the global scale using IPCC Tier 1 guidelines for anaerobic digestion, EPA estimates for fossil fuel emission offsets from energy produced through anaerobic digestion, and our LCA model for biochar-composting.^58,62^ Due to the logistical and economic barriers facing small-scale dairies, we limit our analysis to the number of dairy cows kept in intensive systems with at least 100 head.^2,3^ We find that when solid manure is biochar-composted instead of stockpiled, the technical annual GHG mitigation potential nearly doubles, increasing from 154 to 297 Tg CO_2_e yr^–1^ (using 100-year GWPs) (Figure 4a). When using 20-year GWPs, biochar-composting increases the technical annual GHG mitigation from dairies from 409 to 640 Tg CO_2_e yr^–1^. Annual technical CH_4_ mitigation potential increases from 4.54 to 6.13 Tg CH_4_ yr^–1^ when biochar-composting is implemented (Figure 4b). An annual reduction of 6.13 Tg CH_4_ would account for a 26% reduction in total dairy CH_4_ relative to 23.4 Tg CH_4_ yr^–1^, the current GAINS model estimate of CH_4_ emissions from dairies globally.^2^ However, the GAINS model projects annual baseline dairy CH_4_ emissions to increase by 4.5 Tg CH_4_ yr^–1^ to a total of 27.9 Tg CH_4_ yr^–1^.^2^ This is primarily due to the growth of the dairy industry in developing regions where a lack of effective policy and/or socioeconomic barriers to implementing technical mitigation strategies may limit CH_4_ reduction potential.^2,3,69^ In order to offset increases in dairy CH_4_ from developing countries, developed countries will likely need to ramp up the implementation of manure CH_4_ mitigation strategies along with techniques to reduce enteric fermentation, such as improved feed quality and feed additives, and at the same time, encourage the adoption of low-dairy diets.^3,70^

While our analysis shows the benefit of biochar-composting relative to digester-only systems, there are also large uncertainties associated with this estimate due to a ±30% uncertainty in emission factors when using Tier 1 guidelines as well as a ±20% uncertainty when estimating livestock population.^58^ Assumptions made about the proportion of manure in liquid and solid systems are also a source of uncertainty as this ratio can vary greatly depending on the region.^5^

Other estimates of global livestock manure CH_4_ mitigation are also highly variable and depend largely on model assumptions. For example, CH_4_ mitigation potential estimates by Höglund-Isaksson et al. and Frank et al. for 2030, the deadline to meet the Global Methane Pledge, are lower at 1.21 and 1.43–3.57 Tg CH_4_ yr^–1^, respectively, while Beach et al. predict a much larger reduction of 9.64 Tg CH_4_ yr^–1^.^2,71,72^ The EPA estimates a manure CH_4_ reduction potential for the U.S. dairy industry of 1.64 Tg CH_4_ yr^–1^, which is larger than some of the estimates for global livestock manure mitigation.^62^ Despite the range in estimates, each of these models assumes anaerobic digestion to be the sole manure management strategy. Our analysis suggests that when biochar-composting is combined with anaerobic digestion, the maximum technical manure CH_4_ mitigation potential could increase significantly. While the maximum economic mitigation potential will likely be much lower than our maximum technical mitigation potential due to economic barriers facing dairy farmers, adding biochar-composting to existing anaerobic digestion systems may be a low-cost way to enhance manure CH_4_ mitigation on these farms relative to the high cost of constructing and maintaining digesters.^2,3^ However, the widespread adoption of biochar for use in dairy systems is dependent on a functioning biochar market along with the existence of infrastructure needed to harvest and pyrolyze biomass.^48^

We show that there is substantial additional CH_4_ mitigation potential when solid dairy manure separated from anaerobic digesters is biochar-composted, instead of stockpiled. Incorporating this novel strategy into CH_4_ mitigation models could increase maximum mitigation potentials from the livestock sector and provide governments with an additional strategy to help meet CH_4_ reduction targets. Despite the potential climate benefits of biochar-composting, significant additional cuts to livestock and dairy CH_4_ are likely needed if animal agriculture is to contribute its fair share to the 30% reduction in total CH_4_ required by signees of the Global Methane Pledge. While growth in the dairy industry has slowed in developed countries, it is expected to continue to rapidly expand in developing countries where widescale adoption of manure management practices may be less likely.^2,3,69−73^ To ensure that global dairy CH_4_ emissions decrease over time, developed countries will likely need to further reduce their dairy consumption, in addition to implementing mitigation strategies that target both solid and liquid manure and enteric fermentation.

## Supporting Information Available

Gas sampling design; SEM biochar images; biogenic and 20-year GWP LCAs; cumulative N_2_O; compost NO_3_^–^ and NH_4_^+^; cumulative GHGs; daily GHG fluxes; compost temperature and moisture; CH_4_ and moisture/porosity correlation; life-cycle system boundary; Sankey diagram of carbon flows; direct emission LCA; sensitivity analysis; uncertainty analysis; feedstock/compost/biochar characteristics; CH_4_ model statistics; parameters for sensitivity/uncertainty analyses; values and equations for anaerobic digestion and lagoon emission estimation (PDF)
