# Correction to “Dairy Manure Co-composting with Wood Biochar Plays a Critical Role in Meeting Global Methane Goals”

**DOI:** 10.1021/acs.est.2c06350

**Published:** 2022-09-15

**Authors:** Brendan P. Harrison, Si Gao, Melinda Gonzales, Touyee Thao, Elena Bischak, Teamrat Afewerki Ghezzehei, Asmeret Asefaw Berhe, Gerardo Diaz, Rebecca A. Ryals

Unfortunately, we detected an error in our calculation of cumulative greenhouse gas emissions from our composting experiment. The error occurred during the conversion of cumulative gas emission in terms of wet feedstock to dry feedstock. Unfortunately, we made a mistake in this conversion as we used the wrong moisture content of the initial feedstocks.

This mistake impacted only the value of the cumulative gas emissions for the treatment and control, which are both too low by a factor of ∼2. The correct cumulative greenhouse gas emissions from the manure-only pile are as follows: 5.03 g CH_4_ kg^–1^ dry feedstock, 451 g CO_2_ kg^–1^ dry feedstock, and 0.060 mg N_2_O kg^–1^ dry feedstock. The correct cumulative greenhouse gas emissions from the biochar-compost pile are as follows: 0.81 g CH_4_ kg^–1^ dry feedstock, 280 g CO_2_ kg^–1^ dry feedstock, and 0.119 mg N_2_O kg^–1^ dry feedstock.

There is also a slight difference in cumulative methane reduction compared to that in the original article. The biochar-compost treatment reduced cumulative methane by 83.9% relative to the control, rather than the 79% reduction reported in the original article. This difference in methane reduction does not affect any of our work’s main findings, discussion, or conclusions. Our life-cycle assessment and scaling-up analyses remain unaffected by this error, as they were calculated without the incorrect moisture contents.

Figure 1 has been corrected. We have also corrected cumulative emissions reported in Supplementary Figures 4, 6, 7, and 9.

**Figure 1:**
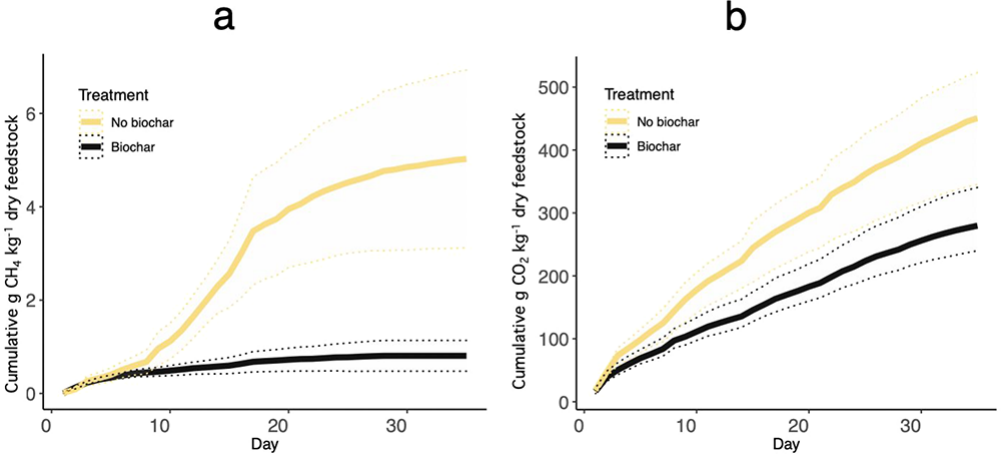
Cumulative (a) CH_4_ and (b) CO_2_ emissions over the 35-day composting experiment. CH_4_ emissions are expressed in units of g CH_4_ kg^–1^ dry feedstock. CO_2_ emissions are expressed in units of g CO_2_ kg^–1^ dry feedstock. The black curve shows cumulative emissions from biochar composting, and the yellow curve shows cumulative emissions from composting. The shaded region for each curve shows the 95% confidence interval for each pile’s gas flux measurements.

## Supporting Information Available

Gas sampling design; SEM biochar images; biogenic and 20-year GWP LCAs; cumulative N_2_O; compost NO_3_^–^ and NH_4_^+^; cumulative GHGs; daily GHG fluxes; compost temperature and moisture; CH_4_ and moisture/porosity correlation; life-cycle system boundary; Sankey diagram of carbon flows; direct emission LCA; sensitivity analysis; uncertainty analysis; feedstock/compost/biochar characteristics; CH_4_ model statistics; parameters for sensitivity/uncertainty analyses; values and equations for anaerobic digestion and lagoon emission estimation (PDF)

