# Supplementary material for: Dairy Manure Co-composting with Wood Biochar Plays a Critical Role in Meeting Global Methane Goals
Source: Environ Sci Technol. 2022 Jul 14;56(15):10987–96. doi: 10.1021/acs.est.2c03467 (PMC9352309; doi:10.1021/acs.est.2c03467)
Supplement: Supplementary file 1 — es2c03467_si_001.pdf [file es2c03467_si_001.pdf]

Supplementary Information for:

Title: Dairy manure co-composting with wood biochar plays a critical role in meeting global methane goals

Authors: Brendan P. Harrison[1], Si Gao[2], Melinda Gonzales[1], Touyee Thao[1], Elena Bischak[1], Teamrat Afewerki Ghezzehei[2], Asmeret Asefaw Berhe[2], Gerardo Diaz[3], Rebecca A Ryals[2]

Affiliation: [1]Environmental Systems Graduate Group, School of Engineering, University of California, Merced, Merced, CA, USA. [2]Department of Life and Environmental Systems, School of Natural Sciences, University of California, Merced, Merced, CA, USA. [3]Department of Mechanical Engineering, School of Engineering, University of California, Merced, Merced, CA, USA.

***Corresponding author:** Brendan Harrison, email: bharrison4@ucmerced.edu

Contents for supplementary information: 21 pages, including 14 supplementary figures and five supplementary tables.

Page contents

1- Cover
2- Sup. Figure 1. GHG sampling design for compost piles
3- Sup. Figure 2. Scanning electron microscopy (SEM) images of biochar
4- Sup. Figure 3. Biogenic and 20-year GWP LCA models
5- Sup. Figure 4. Cumulative N_2O emissions
6- Sup. Figure 5. Compost experiment weekly NH_4^+ and NO_3^-
7- Sup. Figure 6. Weekly cumulative composting GHG emissions
8- Sup. Figure 7. Daily composting GHG fluxes
9- Sup. Figure 8. Compost experiment temperature and moisture
10- Sup. Figure 9. Correlation between CH_4 and moisture/porosity
11- Sup. Figure 10. Life-cycle system boundary
12- Sup. Figure 11. Sankey diagrams of C flows from biochar-composting and composting
13- Sup. Figure 12. 100-year and 20-year GWP direct emission LCA models
14- Sup. Figure 13. LCA global sensitivity analysis
15- Sup. Figure 14. LCA uncertainty analysis
16- Sup. Table 1. Fresh manure, compost, and biochar-compost characteristics
17- Sup. Table 2. Biochar characteristics
18- Sup. Table 3. Model statistics for CH_4 flux
19 & 20- Sup. Table 4. Parameter ranges used in sensitivity and uncertainty analyses
21- Sup. Table 5. Values and equations used to estimate manure CH_4 emissions

Supplementary Figure 1. GHG sampling design for daily compost pile measurements. GHGs were sampled from the North side, South side, and top of each pile at three sections for a total of nine measurements (P1, P2, P3). The nine sampling locations are marked by a white circle with a corresponding number. The pile was approximately 30 m long, 3 m wide and 1 m tall.

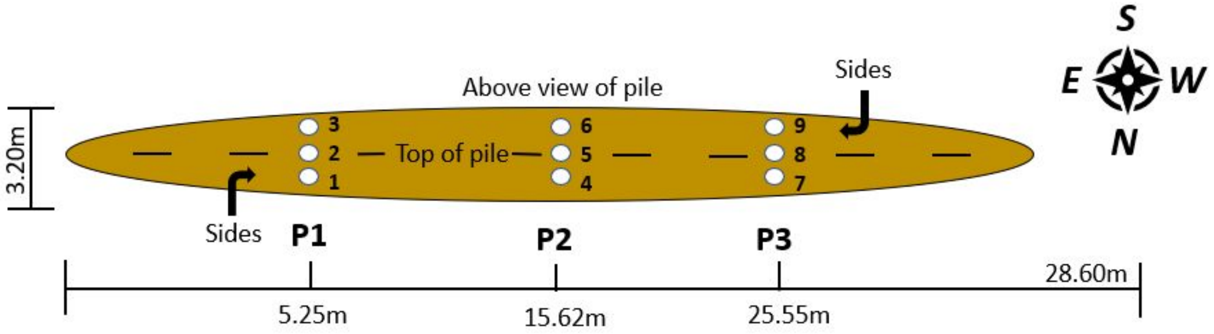

Supplementary Figure 2. **a.** Low magnification (50x) biochar surface image taken through scanning electron microscopy (SEM). **b.** High magnification (200x) biochar surface image taken through SEM.

a b

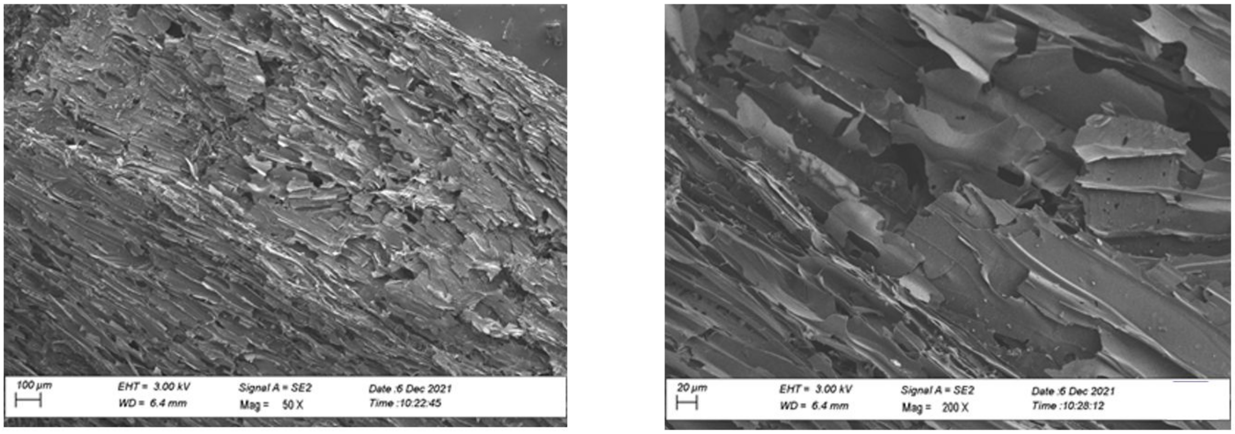

Supplementary Figure 3. a. Life-cycle assessment of management strategies for separated solid dairy manure using 100-year GWPs and including biogenic CO_2 emissions. **b.** Life-cycle assessment of management strategies for separated solid dairy manure using 20-year GWPs. The number above each strategy is the net GWP in kg CO_2e Mg^{-1} manure. Each color represents a different life-cycle stage and is referenced in the legend. The transportation stages are removed from the figures due to their minuscule contribution to the total GWP of each strategy.

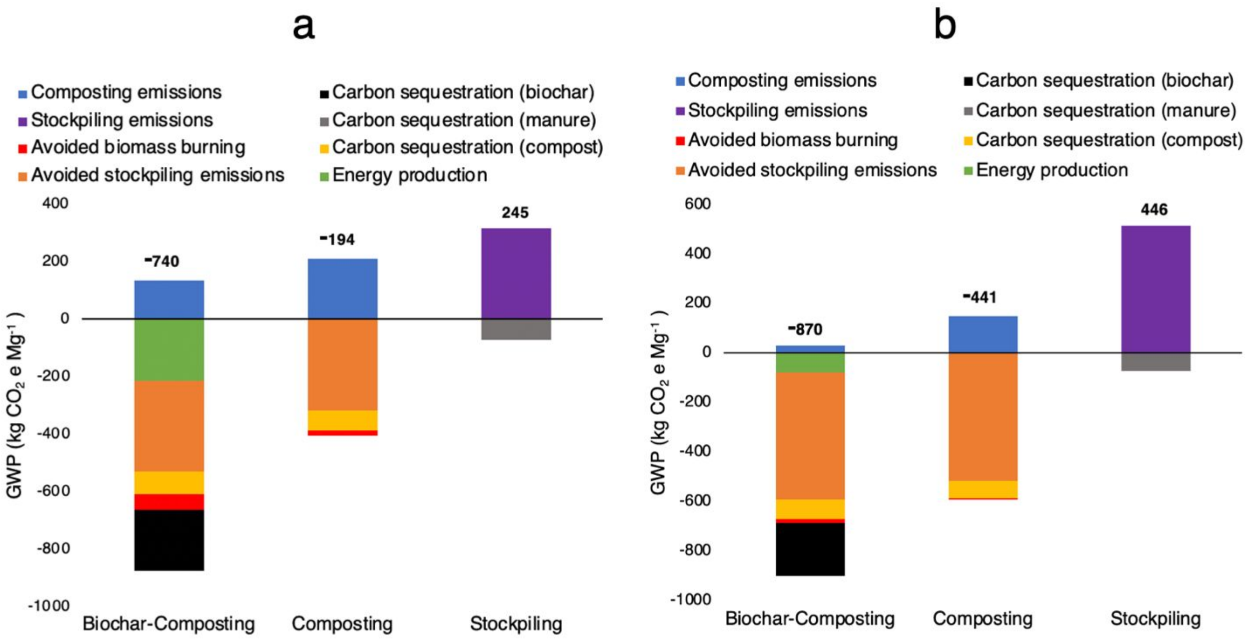

Supplementary Figure 4. Cumulative N_2O emission (mg N_2O kg^{-1} dry feedstock) in a 35-d dairy manure composting field experiment with or without biochar treatment. Shaded area represents 95% confidence interval.

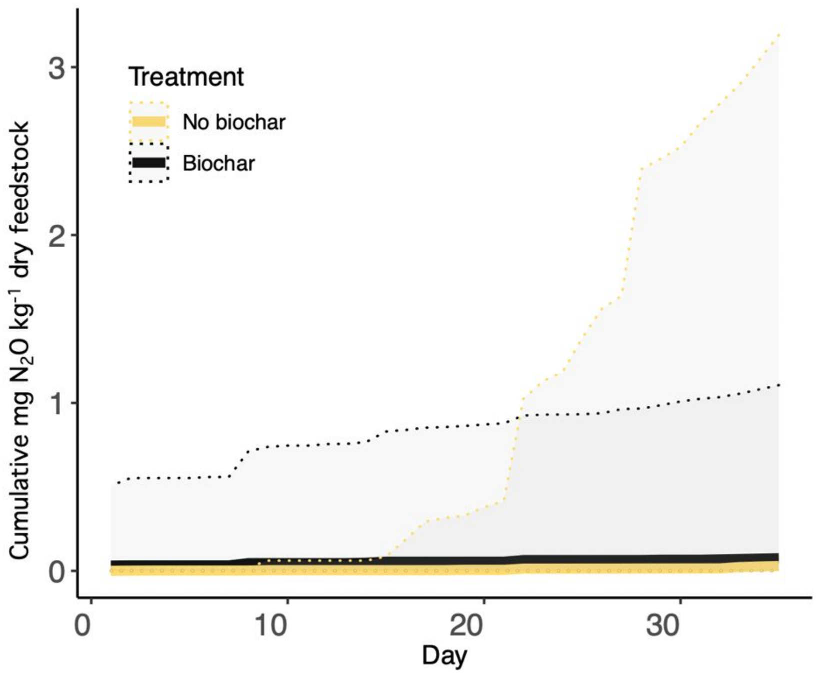

Supplementary Figure 5. Compost (a) NH_4^+ and (b) NO_3^- concentrations (mg N g^{-1}) on Day 1, 7, 14, 21, 28, and 35 in a 35-d dairy manure composting field experiment with or without biochar treatment. Data are presented as average ± 1x standard error (n = 3).

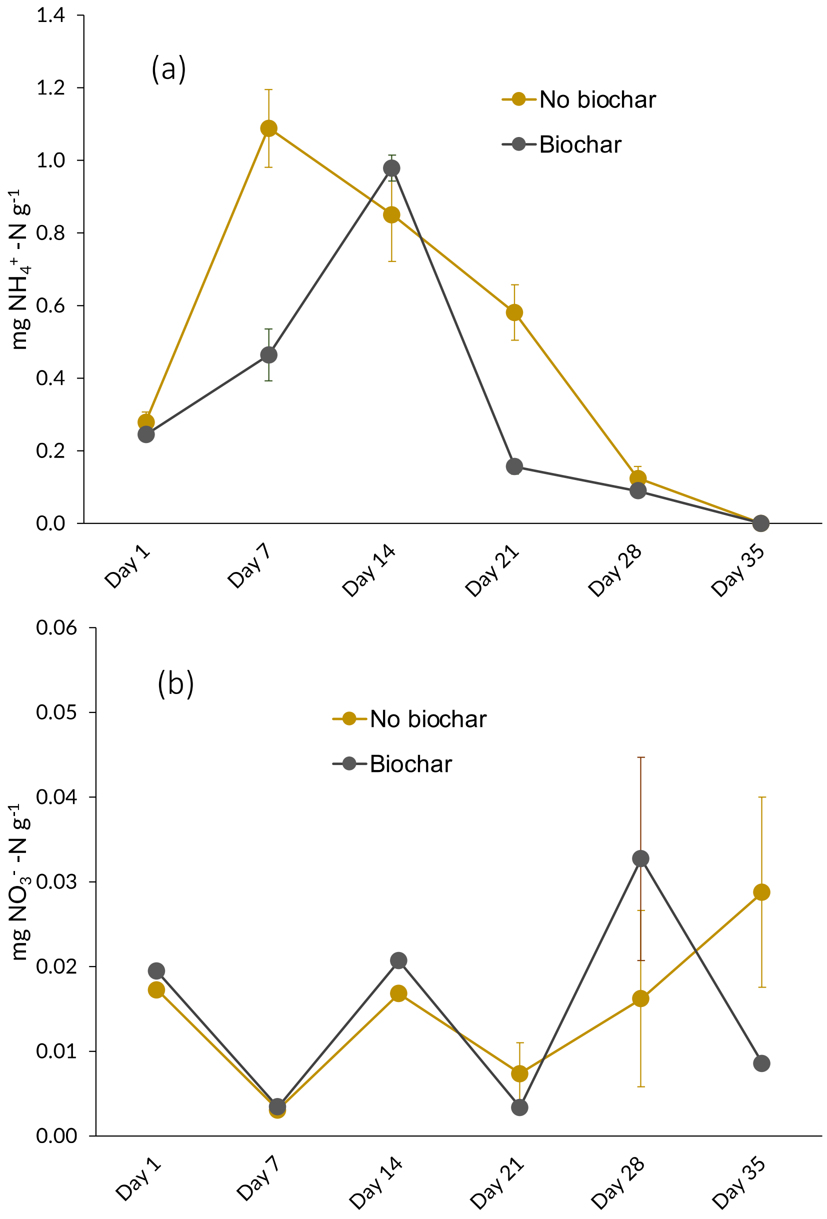

Supplementary Figure 6. Weekly emission of (a) CH_4 (mg C kg^{-1} dry feedstock), (b) CO_2 (g C kg^{-1} dry feedstock), and (c) N_2O (mg N kg^{-1} dry feedstock) in a 35-d dairy manure composting experiment with or without biochar treatment. Data are presented as average ± 1x standard error. Data were compared using Tukey-HSD test following ANOVA. Bars with the same letter were not significantly different at *P* = 0.05.

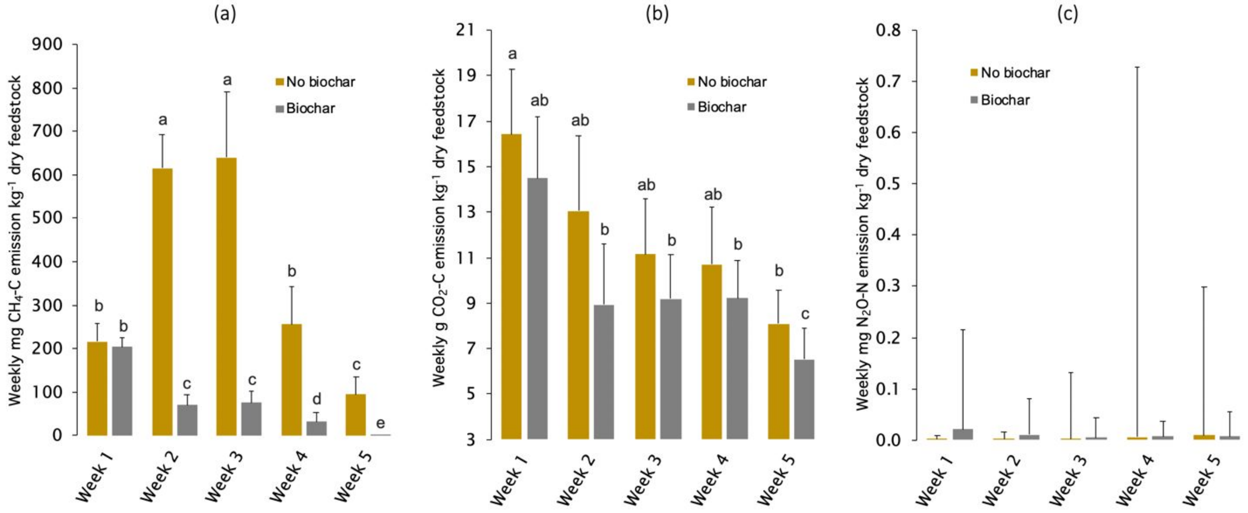

Supplementary Figure 7. Daily (a) CH_4 (mg CH_4-C kg^{-1} dry feedstock), (b) CO_2 (g CO_2-C kg^{-1} dry feedstock), and (c) N_2O (mg N_2O-N kg^{-1} dry feedstock) emissions in a 35-d dairy manure composting field experiment with or without biochar treatment. Data are presented as average ± 1x standard error.

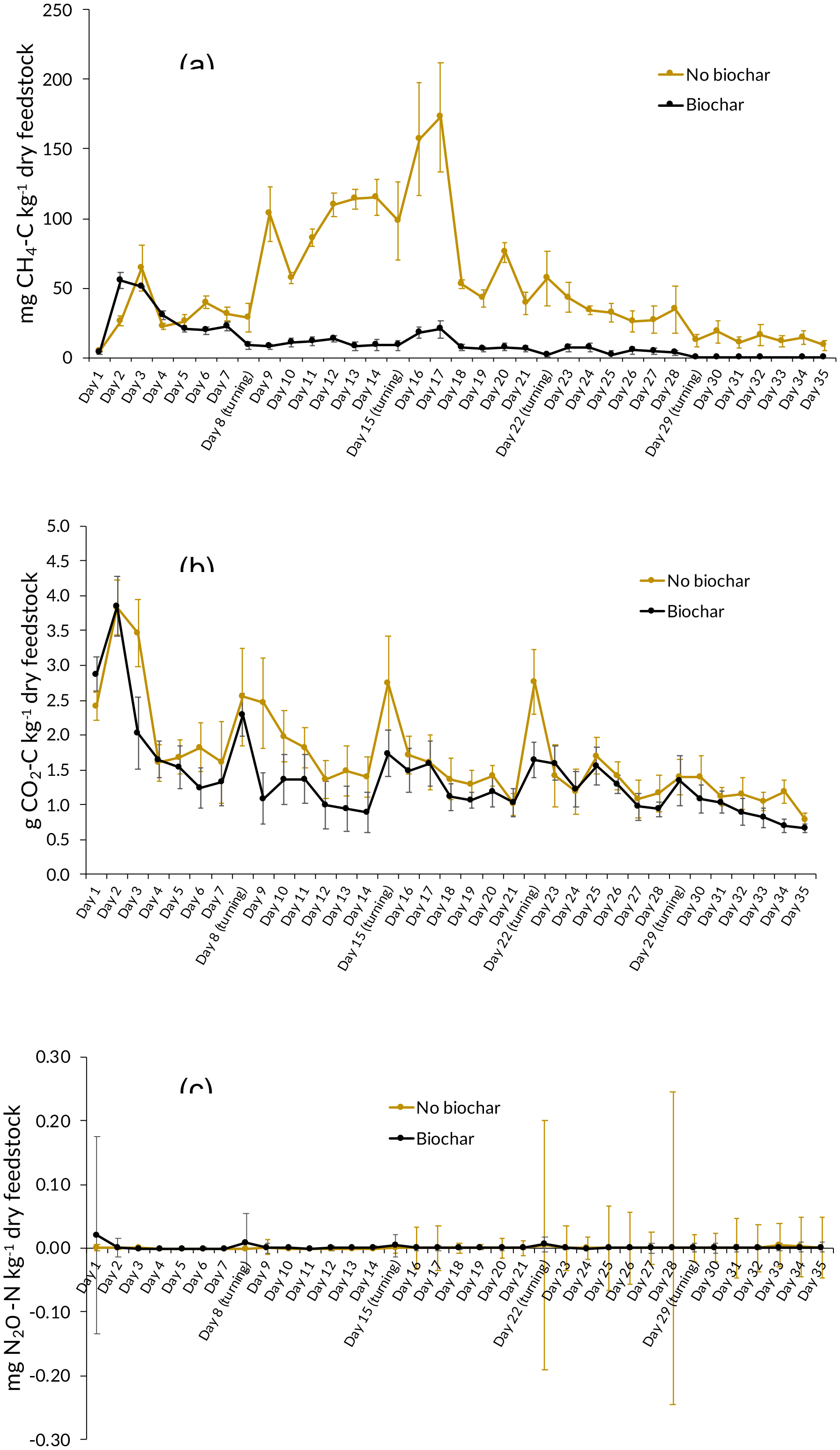

Supplementary Figure 8. Temperature (˚C) and moisture content (%) in a 35-d dairy manure composting field experiment with or without biochar treatment. Turning events occurred on day 8, 15, 22, and 29.

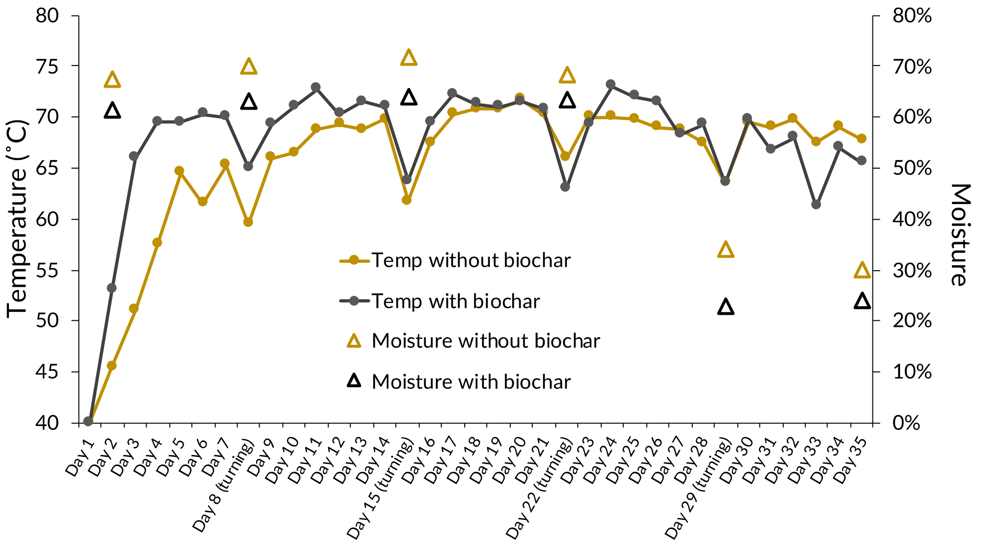

Supplementary Figure 9. Correlation between weekly average CH_4 emission (mg CH_4 kg^{-1} dry feedstock d^{-1}) and (a) weekly pile moisture (Pearson r = 0.56, $P < 0.05$) or (b) weekly pile porosity (Pearson r = 0.10, $P > 0.05$) in a 35-d dairy manure composting field experiment with or without biochar treatment. Weekly average CH_4 emission was calculated by averaging seven daily CH_4 emissions in a week (n=7). Error bar represents 1x standard error.

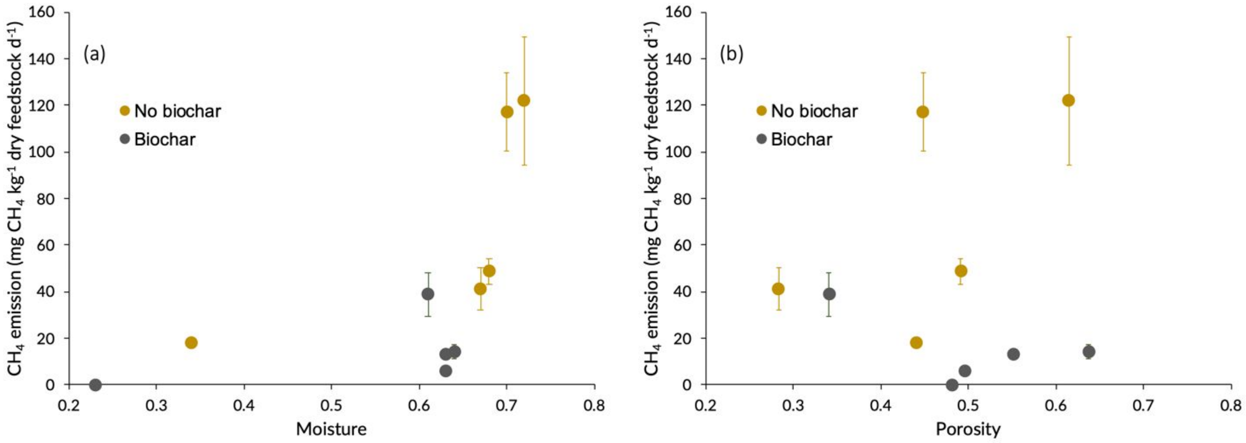

Supplementary Figure 10. Life-cycle system boundaries for each of the three solid manure managements strategies considered in the life-cycle assessment. Ecosystem impacts (e.g. soil N_2O flux, yield increase, etc.) from applying each organic amendment are excluded from each life-cycle assessment due to limited data

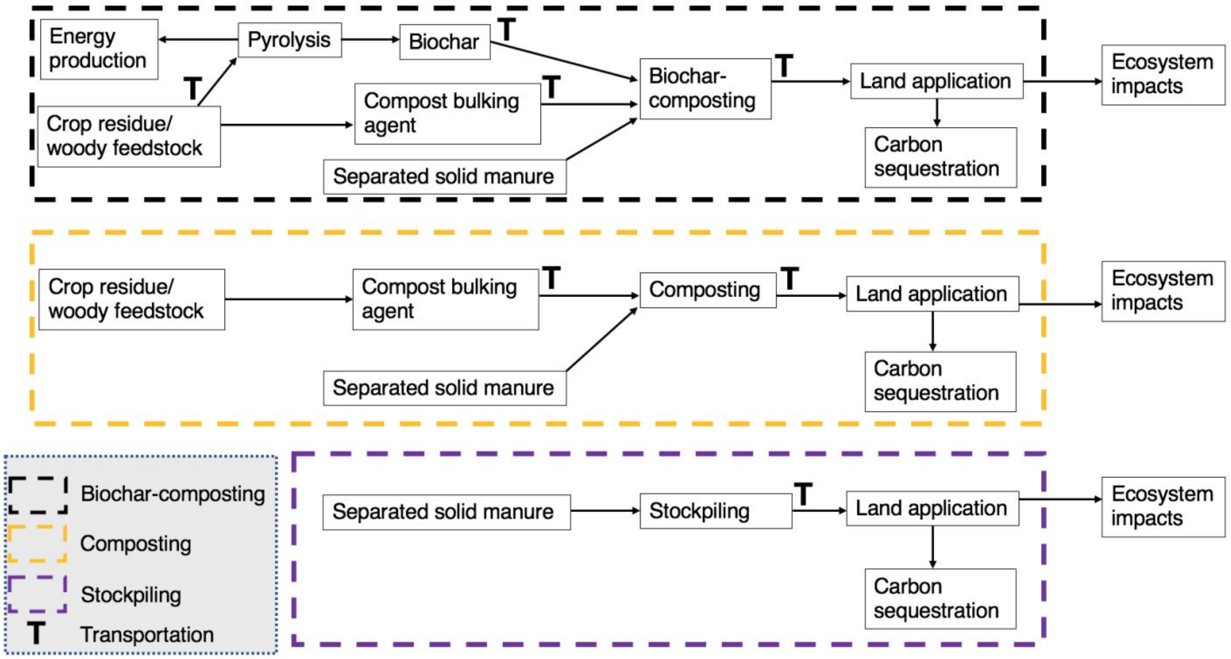

Supplementary Figure 11. Comparative Sankey diagram showing carbon (C) flows from (a) 1 kg of biochar-compost feedstock C and (b) 1 kg of compost feedstock C. Results are based on the proportion of CO_2-C and CH_4-C lost as emissions from biochar-compost and manure-only compost piles during the composting experiment and on the C sequestration rates used in the life-cycle assessment.

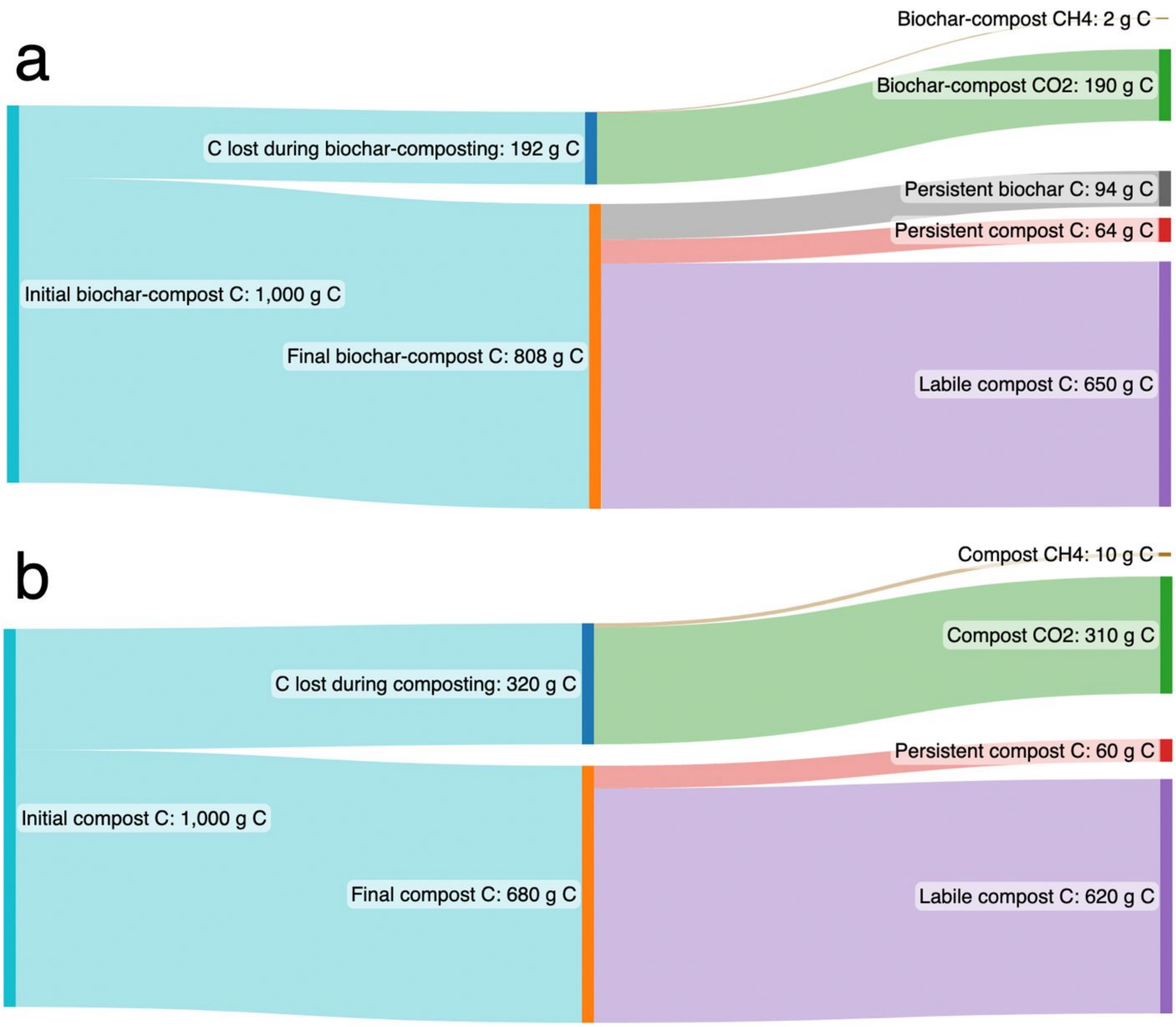

Supplementary Figure 12. a. Life-cycle assessment of management strategies for separated solid dairy manure using 100-year GWPs and including only direct emissions (avoided emissions are excluded). **b.** Life-cycle assessment of management strategies for separated solid dairy manure using 20-year GWPs and including only direct emissions (avoided emissions are excluded). The number above each strategy is the net GWP in kg CO_2e Mg^{-1} manure. Each color represents a different life-cycle stage and is referenced in the legend. The transportation stages are removed from the figure due to their minuscule contribution to the total GWP of each strategy.

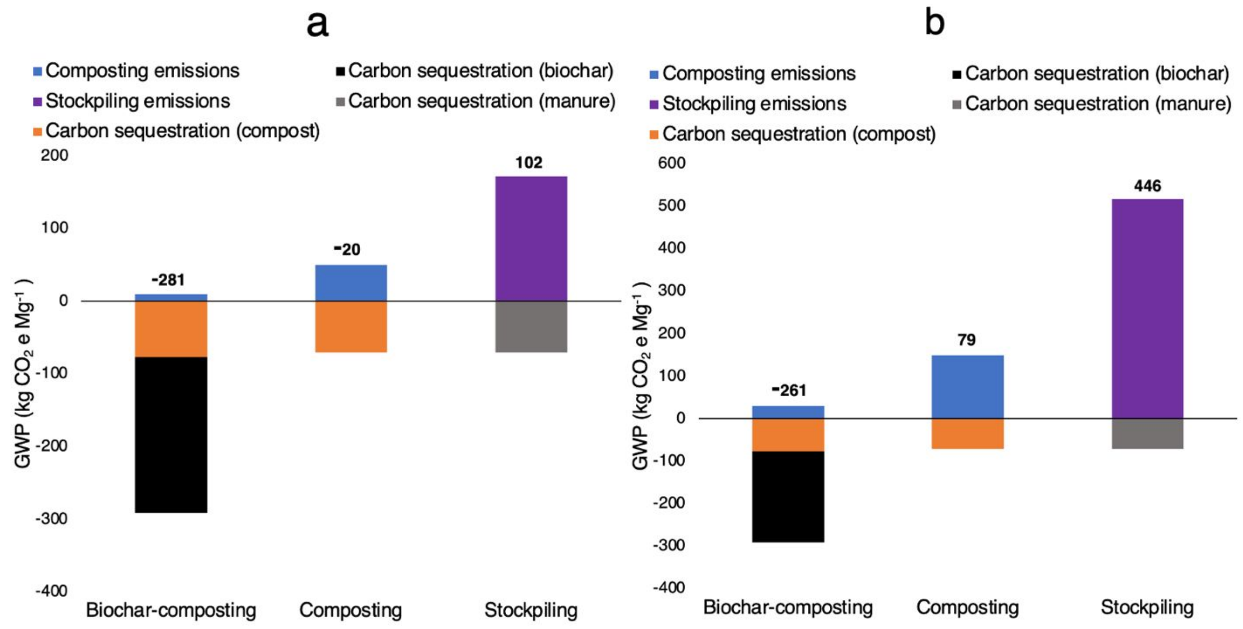

Supplementary Figure 13. Global sensitivity analysis of the LCA for each management strategy. “ST” represents the total order sensitivity of each parameter in the given scenario, and “S1” represents the first order sensitivity. The value of the index is expressed as a percentage of the attributable variance in the output. “B” = biochar-composting, “C” = composting, “SP” = stockpiling, “Trans” = transportation, “C Seq” = manure carbon sequestration, “Av” = avoided, “WB” = woody biomass burning, “Com” = compost, “EF” = emissions factor, “GWP” = global warming potential, “Energy” = energy production from pyrolysis.

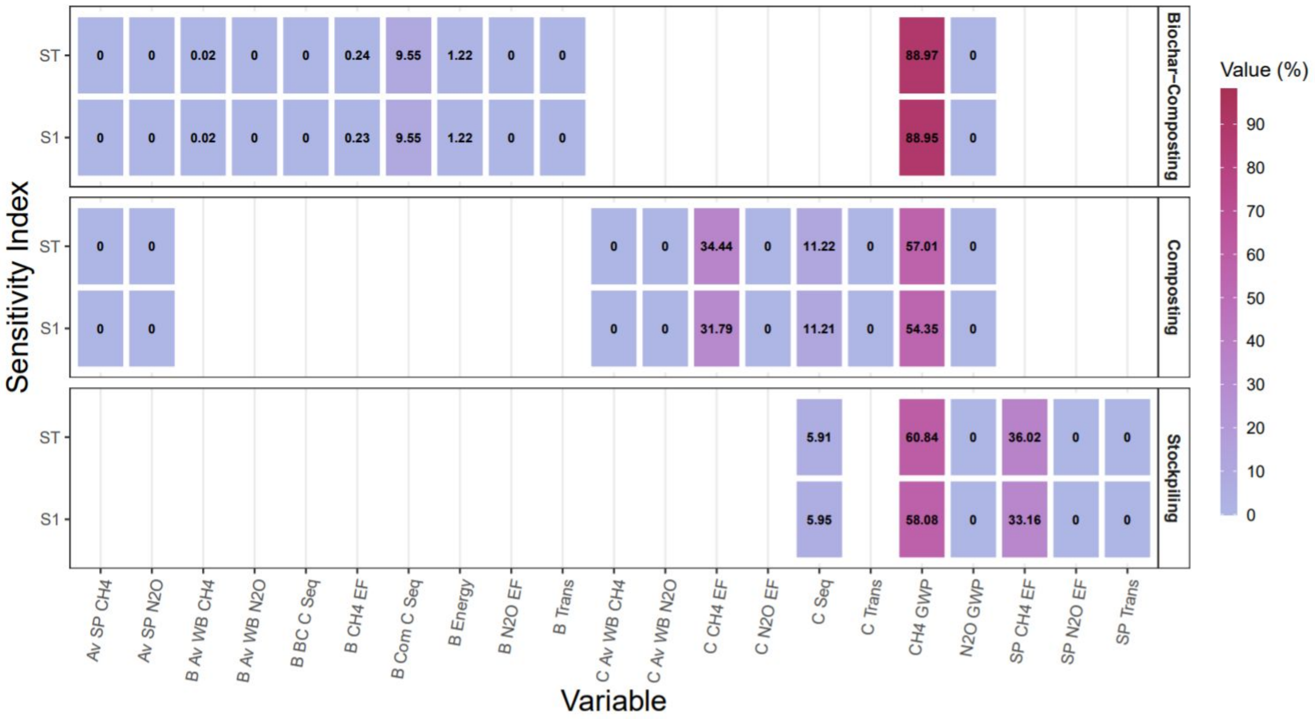

Supplementary Figure 14. Uncertainty of management strategies contribution to net GWP. Results are expressed in kg CO_2e Mg^{-1} manure. Positive numbers indicate net emissions and negative numbers indicate net mitigation.

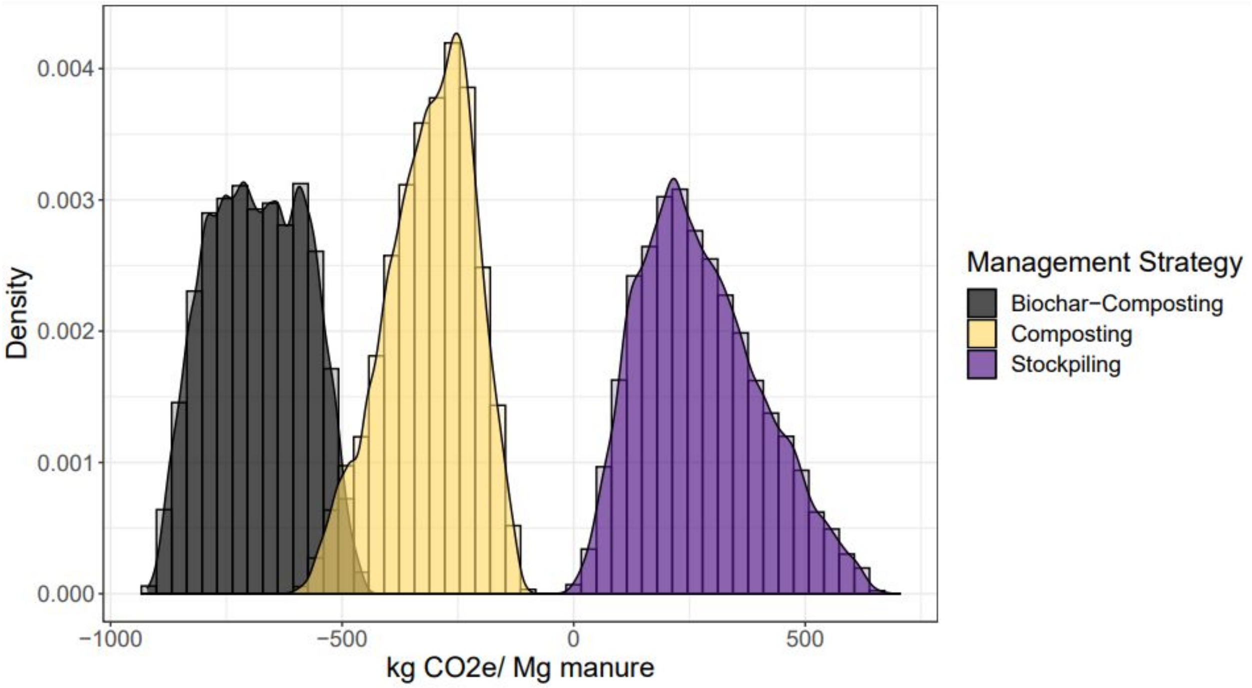

Supplementary Table 1. Characteristics of dairy manure feedstock, compost, and biochar-compost in a 35-d dairy manure composting experiment with or without biochar.

**Please note: We caution against using C/N ratio as a maturity index for biochar-compost as the high C in biochar may result in a high C/N ratio, but this does not necessarily mean a biochar-compost is immature.*

	Fresh manure	Compost (final)	Biochar-compost (final)
Total C (g kg^{-1})	307	394	458
Total N (g kg^{-1})	20.2	21.6	19.4
C/N	15.2	18.2	23.6
pH	7.8	7.5	8.0
CEC (meq /100 g compost)	-	134.8	140.4
EC (dS m^{-1})	2.10	3.00	2.01
Germination index		128	133
Bulk density (g cm^{-3})	0.37	0.38	0.25
Moisture content (fresh wt. %)	78	30	24
Volatile matter (dry wt. %)	86.2	75.0	81.5
Ash (dry wt. %)	13.96	15.68	24.61
Fixed C (dry wt. %)	n.d.	n.d.	0.35
Porosity	0.377	0.491	0.496
NH_4^+-N (mg kg^{-1} dry)	223.5	55.9	10.6
NO_3^--N (mg kg^{-1} dry)	6.52	258.4	219.2
Total P (mg kg^{-1} dry)	-	2200	2400
P_2O_5 (mg kg^{-1} dry)	-	5000	5500
Total K (mg kg^{-1} dry)	-	6300	7100
K_2O (mg kg^{-1} dry)	-	7590	8550
S (mg kg^{-1} dry)	-	2700	2700
Mg (mg kg^{-1} dry)	-	4600	4700
Ca (mg kg^{-1} dry)	-	15100	15300
Na (mg kg^{-1} dry)	-	1100	1500
Fe (mg kg^{-1} dry)	-	2907	2868
Al (mg kg^{-1} dry)	-	1907	1955
Mn (mg kg^{-1} dry)	-	121	153
Cu (mg kg^{-1} dry)	-	29	36
Zn (mg kg^{-1} dry)	-	90	94
B (mg kg^{-1} dry)	-	53	63

Supplementary Table 2. Characteristics of the biochar used in the biochar-composting experiment.

Total C (g kg^{-1})	790
Total N (g kg^{-1})	1.92
H:C (molar ratio)	0.102
O:C (molar ratio)	0.068
Particle size range (mm)	1-4 mm
pH	9.2
EC (dS m^{-1})	1.21
Bulk density (g cm^{-3})	0.08
Moisture content (fresh wt. %)	8.97
Volatile matter (dry wt. %)	55.61
Ash content (dry wt. %)	4.43
Fixed carbon content (dry wt. %)	39.96
NH_4^+-N (mg kg^{-1} dry)	Below instrument detection
NO_3^--N (mg kg^{-1} dry)	3.51
BET surface area (m^2 g^{-1})	437.17
Total pore volume (cm^3 g^{-1})	0.2549
Sorption average pore size (diameter in nm)	2.3
Cumulative surface area of pores between 0.3-1.34 nm hydraulic radius (m^2 g^{-1})	619.15
Cumulative pore volume of pores between 0.3-1.34 nm hydraulic radius (cm^3 g^{-1})	0.245

Supplementary Table 3. Model statistics for CH_4 flux regressed against environmental and biogeochemical factors examined in this 35-d field composting study in a mixed linear model following backward variable selection (model fit R-squared = 0.845, $P < 0.001$). All data were log transformed in the model to ensure data normality and homogeneity of variance. Significant levels: *$P < 0.05$, ** $P < 0.01$, *** $P < 0.001$, ns indicates $P > 0.05$.

Coefficients	t-value	P-value	Level of significance
Intercept	0.501	0.62	ns
Biochar treatment	4.420	3.96e-05	***
Moisture	9.610	5.77e-14	***
Porosity	-5.293	1.62e-5	***
Time since composting	4.561	2.41e-5	***
CO_2 flux	3.294	0.001625	**
NO_3^- -N	3.732	0.000411	***

Supplementary Table 4: Ranges of values used in the sensitivity and uncertainty analysis for each management strategy. "Mid" values were used in the baseline LCA models.

**Parameter**	**Reference**	**Min**	**Mid**	**Max**	**Units**
Stockpiling CH_4 EF	Pardo et al 2015	3.82308846	6.146926537	8.47076462	g CH_4 / kg manure
Stockpiling N_2O EF	Pardo et al 2015	-0.0010408	4.13043E-05	0.00467609	g N_2O / kg manure
Stockpiling CH_4 GWP	IPCC	28		84	g CO_2e/ g CH_4
Stockpiling N_2O GWP	IPCC	264		265	g CO_2e / g N_2O
Manure C seq.	Martinez-Blanco et al., 2013	-15.673913	-70.55434783	-125.43478	g CO_2e / kg manure
Manure transportation	DeLonge et al. 2013, exp data	0.0745	0.149	0.298	g CO_2e / kg manure
**Parameter**	**Reference**	**Min**	**Mid**	**Max**	**Units**
Composting CH_4 EF	exp. data	1.10869565	1.782608696	2.45652174	g CH_4 / kg manure
Composting N_2O EF	exp. data	-0.0020815	2.06522E-05	0.00233804	g N_2O / kg manure
Avoided Stockpiling CH_4	Pardo et al 2015; exp. data	-3.8230885	-6.146926537	-8.4707646	g CH_4 / kg manure
Avoided Stockpiling N_2O	Pardo et al 2015; exp. data	0.00104076	-4.13043E-05	-0.0046761	g N_2O / kg manure
Composting CH_4 GWP	IPCC	28		84	g CO_2e/ g CH_4
Composting N_2O GWP	IPCC	264		265	g CO_2e / g N_2O
Biomass burning CH_4	Andreae, 2019	-0.037913	-0.049565217	-0.0612174	g CH_4 / kg manure
Biomass burning N_2O	Andreae, 2019	-0.0007565	-0.000782609	-0.0008087	g N_2O / kg manure
Compost C seq.	Martinez-Blanco et al., 2013	-15.673913	-70.55434783	-125.43478	g CO_2e / kg manure
Compost transportation	DeLonge et al. 2013, exp. data	0.08744565	0.174891304	0.34978261	g CO_2e / kg manure
**Parameter**	**Reference**	**Min**	**Mid**	**Max**	**Units**

Biochar-composting CH4 EF	exp. data	0.20857474	0.359212051	0.50984936	g CH4 / kg manure
Biochar-composting N2O EF	exp. data	-0.0006779	5.33024E-05	0.00078447	g N2O / kg manure
Avoided stockpiling CH4	Pardo et al., 2015; exp. data	-3.8230885	-6.146926537	-8.4707646	g CH4 / kg manure
Avoided Stockpiling N2O	Pardo et al., 2015; exp. data	0.00104076	-4.13043E-05	-0.0046761	g N2O / kg manure
Biochar-composting CH4 GWP	IPCC	28		84	g CO2e/ g CH4
Biochar-composting N2O GWP	IPCC	264		265	g CO2e / g N2O
Biomass burning CH4	Andreae, 2019	-0.1465122	-0.191541136	-0.2365701	g CH4 / kg manure
Biomass burning N2O	Andreae, 2019	-0.0029235	-0.003024334	-0.0031251	g N2O / kg manure
Biochar-composting compost C seq.	Martinez-Blanco et al., 2013	-15.677868	-70.5561993	-125.42294	g CO2e / kg manure
Biochar-composting biochar C seq.	Wang et al., 2015	-200.61414	-201.8308227	-203.03592	g CO2e / kg manure
Gasification energy production	Roberts et al., 2009; IPCC, 2006	-56.639629	-76.24565469	-95.840093	g CO2e / kg manure
Biochar-compost transportation	Roberts et al., 2009; exp data	0.16483198	0.330359212	0.66071842	g CO2e / kg manure

Supplementary Table 5. Values and equations used to estimate manure CH_4 emissions from anaerobic lagoons and anaerobic digestion for California and global scaling-up analyses. MCF = methane conversion factor. VS_{prod} = Manure volatile solids produced. B_0 = Maximum methane production capacity. EF = emission factor. TAM = Total animal mass.

Analysis	Variable	Value	Reference
California Tier 2 estimation	Anaerobic lagoon MCF (-)	0.748	CARB, 2014
	Anaerobic digestion MCF (-)	0.181	CARB, 2014
	VS_{prod} (kg VS yr^{-1} hd^{-1})	2,833	CARB, 2014
	B_0 (m^3 CH_4 kg VS^{-1})	0.24	CARB, 2014
Tier 2 equation to estimate CH_4 from a manure management strategy	CH_4 emissions (kg CH_4 hd^{-1} yr^{-1}) = MCF * VS * B_0 * 0.622 (m^3 CH_4 / kg CH_4) * fraction manure managed w/ strategy		
Global Tier 1 estimation	Anaerobic lagoon EF (g CH_4 kg VS^{-1})	100.5	IPCC, 2019 (average of cool & temperate climates)
	Anaerobic digestion EF (g CH_4 kg VS^{-1})	3.45	IPCC, 2019 (average of cool & temperate climates)
	VS_{prod} (kg VS 10^3 kg animal $mass^{-1}$ yr^{-1})	2,965.63	IPCC, 2019 (average from N.A., Europe, Asia)
	TAM (kg animal mass / animal)	526	IPCC, 2006 (average from N.A., Europe, Asia)
Tier 1 equation to estimate CH_4 from a manure management strategy	CH_4 emissions (g CH_4 hd^{-1} yr^{-1}) = EF * VS_{prod} * TAM * fraction manure managed w/ strategy		
